# Clinical Presentation and Treatment of Early-Onset Behavior Disorders: The Role of Parent Emotion Regulation, Emotion Socialization, and Family Income

**DOI:** 10.1177/01454455211036001

**Published:** 2021-08-11

**Authors:** April Highlander, Chloe Zachary, Kaeley Jenkins, Raelyn Loiselle, Madison McCall, Jennifer Youngstrom, Laura G. McKee, Rex Forehand, Deborah J. Jones

**Affiliations:** 1 University of North Carolina at Chapel Hill, Chapel Hill, NC, USA; 2 Triangle Area Psychology Clinic, Durham, NC, USA; 3 Georgia State University, Atlanta, GA, USA; 4 University of Vermont, Burlington, VT, USA

**Keywords:** behavioral parent training, emotion socialization, emotion regulation, income, early childhood

## Abstract

Parent emotion regulation and socialization have been linked to various aspects of child functioning. In the case of early-onset behavior disorders in particular, parent emotion regulation may be an important correlate of the coercive cycle implicated in early-onset behavior disorders thus, symptom presentation at baseline. Further, emotion socialization may be complicated by a pattern of parent-child interactions in which both supportive or unsupportive parenting behaviors in response to behavioral dysregulation may increase vulnerability for problem behavior in the future. Some work suggests standard Behavioral Parent Training may impact parent emotion regulation and socialization. Still little is known, however, about how such processes may vary by family income, which is critical given the overrepresentation of low-income children in statistics on early-onset behavior disorders. This study explored parent emotion regulation, socialization, and family income in a sample of socioeconomically diverse treatment-seeking families of young (3–8 years old) children. Findings suggest relations between parental emotion regulation, socialization, and child behavior although the pattern of associations differed at baseline and post-treatment and varied by family income. Clinical implications and future directions are discussed.

Estimates suggest that eight million (16%) U.S. youth have a behavior disorder (BD), including Attention-Deficit/Hyperactivity Disorder (ADHD), Oppositional Defiant Disorder (ODD), and Conduct Disorder (CD) (e.g., August et al., 1996; Ghandour et al., 2019; Larson et al., 2011; Merikangas et al., 2009). Early-onset (3–8 years old) behavior disorders can lead to life-long problems, including delinquency/antisocial behavior, depression/anxiety, substance abuse/dependence, and relationship/employment instability (e.g., Burke et al., 2014; Fergusson et al., 2013; Owens & Hinshaw, 2016). In turn, behavior disorders increase individual health care, education, and criminal justice costs 10-fold by the age of 30 alone (e.g., Pelham et al., 2007; Piquero et al., 2009; Scott et al., 2001), making standard of care, early-intervention Behavioral Parent Training (BPT), a clinical and public health imperative. The evidence-base for BPT, which yields better treatment outcomes than any other treatment approach, is well-established (see Chorpita et al., 2011; Eyberg et al., 2008; Forehand et al., 2013; Furlong et al., 2013; Shelleby & Shaw, 2014). Yet, a quarter to a third of families still fail to benefit from BPT, suggesting the importance of further inquiry into factors posited to impact treatment effectiveness (see Leijten et al., 2018; Lundahl et al., 2006; Shaw, 2013 for reviews). One such line of work has considered parent emotion regulation and socialization.

Definitions vary, however, there is general agreement that emotion regulation includes both extrinsic (e.g., selection and modification of situations that give rise to emotion) and intrinsic (e.g., deployment of attention in response to emotion) processes inherent in monitoring, evaluating, and adapting both positive and negative emotional reactions (see Compas et al., 2015, 2017, for reviews). The transition to parenthood is posited to be associated with neurobiological, hormonal, and psychological changes that foster emotion regulation in preparation for childrearing; yet, variability in regulatory functioning exists both within and between parents’ (see Rutherford et al., 2015 for a review). In the case of early-onset behavior disorders, variability in stress and the way parents regulate their own emotions in the context of stress may heighten risk for the coercive cycle of parent-child interaction implicated in early-onset behavior disorders (Patterson, 1982; Reid et al., 2002; also see Lunkenheimer et al., 2016 for a review). That is, early-onset behavior disorders are thought to evolve through a transactional process whereby temperamentally difficult children engage in behaviors (e.g., aggression, noncompliance, tantrums) that elicit inconsistent and/or negative attention from parents (e.g., negotiating, threatening, yelling), which then inadvertently increases (rather than decreases) the likelihood of subsequent problem behavior (see Chang & Shaw, 2016; Dishion & Snyder, 2016; Forehand et al., 2013 for reviews). While difficult for any parent, more effectively responding to child problem behavior may be most challenging for those parents who have more difficulty experiencing, expressing, and effectively coping with their own emotions (e.g., Breaux et al., 2016; Gross, 2011; Gross & Thompson, 2007; Thompson, 1994). That is, parents of children with early-onset behavior disorders must simultaneously regulate their own emotions and engage in parenting behaviors that help the child regulate in the moment and learn to self-regulate in the future as well. Accordingly, this study examined the interrelationship of parent emotion regulation and child problem behavior at baseline, whether parent emotion regulation changes as a function of participation in BPT, and whether those changes are associated with improved child outcomes.

Parent emotion socialization (Morris et al., 2007), which describes the processes through which a parent models, reacts to, and coaches children’s emotions, has also been considered in the study of early-onset behavior disorders. Specifically, parental emotion socialization, particularly non-supportive emotion socialization behaviors (e.g., minimizing, punishing or distressed reactions to children’s emotions) are posited to maintain and prolong a child’s emotion arousal and, in turn, dysregulated behavior (Eisenberg et al., 1996; also see Eisenberg et al., 1998; Johnson et al., 2017 for reviews). That said, the data also suggests that it is important that parental socialization of emotion is not one-size-fits-all, but rather is tailored to or fits with the disposition and needs of the child (Eisenberg, 2020; Grolnick et al., 1997). This “fit” may be particularly challenging for parents of children with early-onset BDs, given that unsupportive or supportive emotion socialization may serve as parental attention and inadvertently maintain or increase problem behavior. Given this potential challenge, this study also explored the interrelationship of parent emotion socialization and child behavior at baseline, as well as changes in parent emotion socialization and the link between those changes and child outcomes post-treatment.

Intervention work conducted to date on if and how BPT can improve parent emotion regulation and/or socialization is still in a relatively preliminary stage with somewhat mixed findings with regard to problem behavior outcomes in particular (Dunsmore et al., 2016; also see Johnson et al., 2017 for a review). For example, Chronis-Tuscano et al. (2016) reported that the majority of families enrolled in a program that included Parent-Child Interaction Therapy (PCIT; Eyberg & Boggs, 1998) enhanced with an additional module targeting parent emotion coaching and modeling (PCIT-Eco) experienced improvements in child behavior problems. Consistent with the pilot nature of the study, however, they used a small, uncontrolled case series design and did not examine changes in parent emotion regulation or socialization across the program. In another pilot study, Salmon et al. (2014) conducted a randomized control trial comparing Emotion Enhanced Triple P (EETP), which taught parents to use supportive emotion socialization strategies, to standard Triple P. EETP increased parents’ emotion socialization, including use of emotion labels, discussion of emotion causes, and emotion coaching at post-treatment relative to standard Triple P; however, only gains in emotion coaching were preserved at follow-up. Moreover, trends in the data suggested that the addition of the emotion socialization content may have compromised the effectiveness of Triple P on child problem behavior at post-treatment. Both groups looked similar, however, at follow-up.

In light of these relatively preliminary and mixed findings, another question remains: Can standard BPT improve parent emotion regulation and socialization toward improved child outcomes? Importantly, standard BPT does not explicitly address parent emotion regulation or socialization. Standard BPT does, however, teach parents the theory behind the etiology and maintenance of early-onset BDs (including the role of parenting in general and parental attention in particular) and skills to better manage those behaviors. Therefore, standard BPT may indirectly function to improve parent emotion regulation and socialization via a better understanding of “fit” between their own and their child’s behavior and, in turn, to feel more efficacious responding to child behaviors in a more regulated and informed way. To this end, prior work on both PCIT and Helping the Noncompliant Child (HNC; McMahon & Forehand, 2003) suggest the potential for improvements in parent emotion regulation and socialization in standard BPT, including improvements comparable to programs targeting those variables in particular (e.g., Lieneman et al., 2019; Rothenberg et al., 2019; Zachary et al., 2019). Yet, we still know little about how certain contextual factors may impact the ways in which BPT treatment effects parent emotion regulation and socialization. One contextual variable that may be particularly important to consider in this line of work is family income.

Low-income children are overrepresented in statistics on behavior disorders (e.g., D’Onofrio et al., 2009; Piotrowska et al., 2015; Shaw & Shelleby, 2014). Although there are likely direct effects of income on child well-being as well, financial strain also increases the risk for child problem behavior through compromises in parenting (Shaw & Shelleby, 2014). Indeed, stressors have been shown to impact parents’ ability to regulate their own emotions and, in turn, to effectively socialize their children around emotions (e.g., Havighurst & Kehoe, 2017; Lieneman et al., 2019; Shaffer & Obradović, 2017). Some work suggests that greater socioeconomic stress in particular may decrease individuals’ sense of control over their environment and, thus, places a premium on the capacity for self-regulation, including emotion regulation (Troy et al., 2017). Although to our knowledge there has not yet been a study to examine if the links between parent emotion regulation, socialization, and child behavior are shaped by family income, some related work on parent-child interaction quality more generally does suggest that lower SES is associated with poorer parental self-regulation (Blair & Raver, 2012). Yet, other work suggests that while relatively lower SES is associated with impaired inhibitory control it is not associated with parental emotion regulation in particular (Shaffer & Obradović, 2017). These mixed findings may be a function of samples with relatively little variability in fairly well-educated (e.g., only 17% of sample earning HS degree or less) and relatively higher income (e.g., 23% of sample earning less than $50,000) families as the authors note (Shaffer & Obradović, 2017).

## Method

### Participants

The current study represents secondary data analysis of 112 families who enrolled in one evidence-based BPT program, Helping the Noncompliant Child (HNC; McMahon & Forehand, 2003). To be eligible, families had to have a child between 3 and 8 years old who met clinical cut-offs for significant problem behavior as defined by the Problem (15) and/or Intensity (131) Subscales of the Eyberg Child Behavior Inventory (ECBI; Eyberg & Pincus, 1999), a standard measure of disruptive behaviors. Exclusion criteria included *current* parental substance abuse/dependence diagnosis, psychotic disorder diagnosis, or severe depression/manic episode, or child psychotic disorder, mood disorder with psychotic features, or pervasive developmental disorder or other disability that would require significant adaptations to standard BPT (e.g., difficulty understanding verbal praise, inability to follow clear instructions, limitations getting in and out of a Time-Out chair or space). The institutional review board approved all study procedures. Consent was obtained by parents for their own and their child’s participation.

Of the 112 enrolled families, 79 completed treatment including all pre–post treatment assessments and are the focus of these analyses. Children were a mean age of 4.48 years (*SD* = 1.31), approximately half boys (56%), and one-third (36%) racial or ethnic minorities. On average, parents were 36 years (*SD* = 6.54) old, the majority were female (92%), most were married (73%), and were employed at least part-time (62%). Reflecting the diversity within and between families, 22% of parents were a racial or ethnic minority. Families’ gross annual income ranged from $15,600 to $300,000 (*M* = $77,604, *SD* = $73,933). Although families’ income spanned a large range with several outliers, appropriate data analytic techniques were employed in order to include all data in analyses (Aguinis et al., 2013). All demographics can be found in Table 1. Families who dropped out of treatment (*n* = 33) did not differ from completers (*n* = 79) on any study variables used in the current analyses; however, parents who completed treatment were significantly older (*t*(110) = −3.71, *p* < 0.01) and had higher levels of education (*t*(110) = −3.24, *p* < 0.01).

**Table 1.** Demographic Characteristics of the Participating Families.

Measure	*n*	%	*M*	*SD*	Range
Parent sex					
Female	73	92.4			
Male	6	7.6			
Parent age			36	6.54	24–57
Parent race/ethnicity					
White	66	83.5			
Black/African-American	8	10.1			
Asian/Pacific Islander	1	1.3			
More than one race	4	5.1			
Hispanic/Latino	5	6.3			
Parent marital status					
Never married	7	8.9			
Married	58	73.4			
Common law	1	1.3			
Separated	3	3.8			
Divorced	10	12.7			
Income			77,604	73,933	15,600–300,000
Federal poverty level	11	13.9			
Employment status					
Employed	49	62.0			
Unemployed	30	38.0			
Education					
Less than HS/GED	2	2.5			
Some college	12	15.2			
College degree	36	45.6			
Advanced degree	29	36.7			
Child sex					
Female	35	44.3			
Male	44	55.7			
Child age			4	1.31	3–8
Child race/ethnicity					
White	61	77.2			
Black/African-American	7	8.9			
Asian/Pacific Islander	1	1.3			
More than one race	9	11.4			
Hispanic/Latino	12	15.2			
Not reported	1	1.3			

### Procedure

Interested families contacted project staff for a brief phone screen to assess key eligibility criteria (i.e., 3- to 8-year-old child, clinically elevated externalizing problems). Eligible and interested parents then received a more extensive parent assessment in a community clinic, which included parent consent for their own and their child’s participation, child assent, confirmation of eligibility criteria through semi-structured interviewing, and collection of additional information about the family including constructs of interest in the current study (see “Measures” section). Semi-structured diagnostic interviews were completed using the MINI International Neuropsychiatric Interview (Sheehan et al., 1998) and MINI International Neuropsychiatric Interview for Children and Adolescents (MINI-KID; Sheehan et al., 2010) to confirm child and parent eligibility.

Families participated in HNC (McMahon & Forehand, 2003), a mastery-based, two-phase Hanf-Model (Kaehler et al., 2016) BPT program designed to teach parents effective behavioral child management strategies particularly for young children with clinically significant problem behaviors. This program occurs in the context of two phases: Phase I (i.e., increase parent’s positive attention to child behaviors they want to increase) and Phase II (i.e., increase parent’s use of clear instructions and consequences for noncompliance). Throughout each phase, clinicians teach skills and actively coach parents in implementing specific skills (e.g., ignoring, verbal labeled praise) during 50-minute play interactions with their child. Phase I is characterized by the use of Differential Attention where parents implement skills of positive attention using attends (e.g., “You picked up the crayon.”) and rewards (e.g., “Good job picking up the toys!”) ignoring minor problem behavior (e.g., whining), and the elimination of instructions, teaching, and questions in the context of “Child’s Game” (i.e., child-directed play). Phase II of treatment is characterized by Compliance Training where parents are taught to use the “Clear Instruction Sequence” in which they implement clear instructions (e.g., “Please hand me the block.”), a warning statement, and the nonphysical discipline procedure, “Time-Out,” when appropriate. In HNC, Time-Out consists of a 3-minute removal of attention (i.e., not looking at, talking to, or touching the child) where the child is instructed to sit on a Time-Out chair or stay in a designated Time-Out space in the room. If the child exhibits aggression or noncompliance to Time-Out, appropriate back-up procedures are implemented as needed (e.g., removing toys and other objects from the room, additional time spent in Time-Out) and the reintroduction of the Clear Instruction Sequence is given until the child exhibits compliance. In addition to skills practice that occurs during treatment sessions, parents are instructed to practice Phase I skills at home, 15 minutes per day, for the duration of treatment. Successful completion of HNC occurs once parents meet mastery criteria for all skills across Phase I and II, usually requiring a total of 8 to 12 sessions (McMahon & Forehand, 2003).

Post-assessment procedures were similar to baseline and families were compensated with $50 for each assessment. Families were also provided a list of additional community mental health resources to facilitate further opportunities for treatment if they were interested.

### Measures

#### Demographics

Parents reported on a number of demographic variables, including family income. Family income was measured as total annual earnings before taxes (i.e., gross annual income). Using Federal Poverty Limit guidelines, income was counted in the form of money, goods, property, and services. Income includes wages and tips, unemployment, pensions and annuities, income from businesses or personal services, dividends and taxable interest, alimony, and rents and royalties. Not included in income is most social security, child support, gifts, and scholarships.

#### Child problem behavior

Intensity and Problem subscales on the 36-item ECBI (Eyberg & Pincus, 1999) at baseline and change pre-to-post treatment were examined, given the availability of normative data (Burns et al., 1991) and established psychometrics (e.g., Fernandez et al., 2011). Each item prompts parents to rate the intensity of a specific behavior occurring (0 = *never* to 7 = *always*) and whether they consider each behavior to be a problem (0 = *no*; 1 = *yes*). Total scores represent the strength of children’s problem behavior and scores that are two or more standard deviations above the normed mean of each subscale is considered clinically significant (Intensity clinical cutoff = 131; Problem clinical cutoff = 15). Alphas of the current study were 0.91 (Intensity) and 0.80 (Problem).

#### Parent emotion regulation

The Difficulties in Emotion Regulation Scale (DERS; Gratz & Roemer, 2004) was used to measure parents’ emotion regulation (ER). This 36-item measure yields a composite total score as well as scores for the following subscales: (a) Nonacceptance subscale, nonacceptance of negative emotions; (b) Goal subscale, difficulties in engaging in goal-directed behaviors when experiencing negative emotions; (c) Impulse subscale, impulse control difficulties; (d) Strategies subscale, limited access to emotion regulation strategies; (e) Awareness subscale, lack of emotional awareness; and (f) Clarity subscale, lack of emotional clarity. The DERS has high internal consistency (α = .93), good test–retest reliability, adequate construct and predictive validity (Gratz & Roemer, 2004), and is sensitive to change over time (Fox et al., 2008). Total scale scores were used in these analyses (α = .93).

#### Parent emotion socialization

The Coping With Children’s Negative Emotions Scale (CCNES; Fabes et al., 2002) measures parents’ emotion socialization (ES) practices. The CCNES consists of six 12-item subscales that assess parental responses in reaction to young children’s negative emotions: (a) Problem-Focused Reactions, (b) Emotion-Focused Reactions, (c) Expressive Encouragement, (d) Minimization Reactions, (e) Punitive Reactions, and (f) Distress Reactions. In accordance with prior studies evaluating ER and ES in children with Behavior disorders (Denham & Kochanoff, 2002; Zachary et al., 2019), the current study grouped these subscales into two broader domains including non-supportive responses (CCNES Non-supportive, including Distress, Minimization, and Punitive Reactions), and supportive responses (CCNES Supportive including Expressive Encouragement, Emotion-focused, and Problem-focused Responses). Higher levels of non-supportive responses to children’s emotions represent more maladaptive aspects of emotion socialization processes while higher levels of supportive responses represent more adaptive parental ES. Previous research has demonstrated that the CCNES has good internal and test–retest reliability and is sensitive to change over time (e.g., Denham & Kochanoff, 2002; Eisenberg & Fabes, 1994; Herbert et al., 2013). The alphas for the current study are .94 for supportive responses and .85 for non-supportive responses.

## Results

### Plan of Analyses

Given the relatively limited work in this area, results focus on correlations and effect sizes, rather than statistical significance only (e.g., Thompson, 2002, 2006; Zachary et al., 2019). Interpretations of results will be limited to medium and large effect sizes to be most conservative (≤.14 small, 0.15–0.34 medium, and ≥.35 large; Cohen et al., 2013).

### Baseline Associations

First, bivariate associations between baseline child behaviors and income, parental emotion regulation, and socialization were evaluated. Parent’s emotion regulation (DERS) was not associated with child problem behavior at baseline, but was associated with their supportive emotion coaching behaviors (*r* = −.38, *p* < .01). Parents who reported greater emotion regulation also reported more supportive coaching of their children’s emotions (see Table 2). Parents who reported higher non-supportive responses to children’s emotions on the CCNES were more likely to be lower income (*r* = −.26, *p* < .05) and to report higher ECBI Intensity scores (*r* = .24, *p* < .05).

**Table 2.** Correlations of Baseline Parent and Child Measures of Interest and Treatment Efficiency.

Variable	1	2	3	4	5	6	7	8
1. Parental ER (DERS)	—							
2. Parental non-supportive ES (CCNES non-supportive)	.19	—						
3. Parental supportive ES (CCNES supportive)	−.38**	−.48**	—					
4. Income	−.08	−.26*	.07	—				
5. ECBI intensity	−.14	.24*	.08	.00	—			
6. ECBI problem	−.06	.18	.13	.03	.68**	—		
7. Total number of sessions	−.02	−.06	.01	.05	−.05	−.01	—	
8. Weeks to complete treatment	−.01	−.08	.06	.11	.08	.13	.24*	—

*Note.* DERS = Difficulties in Emotion Regulation Scale; CCNES = Coping With Children’s Negative Emotions Scale; ECBI = Eyberg Child Behavior Inventory.

* *p* < .05. **p < .01.

As shown in Table 3, multiple regression analyses were performed to assess the relationship of baseline parent emotion regulation (DERS), emotion socialization (CCNES), and income to baseline child problem behavior (ECBI Intensity and Problem) using partial eta squared (η_ρ_²) as a measure of effect size. Family income and parent DERS scores were not associated with ECBI Intensity or Problem scores at baseline. The effect size of the CCNES Non-supportive for ECBI Intensity (*Β* = 5.60, *p* < .01, η_ρ_² = .32) and Problem (*Β* = 1.01, *p* = .01, η_ρ_² = .29) scores, however, was medium and significant with the pattern of associations suggesting that higher levels of maladaptive parental ES were significantly associated with parents endorsing a greater occurrence and negative perception of problem behaviors at baseline. Additionally, the magnitude of the associations of CCNES Supportive with ECBI Intensity (*Β* = 2.43, *p* = .13, η_ρ_² = .17) and Problem (*Β* = 0.67, *p* < .05, η_ρ_² = .23) scores at baseline were also medium. However, this association was only significant for CCNES Supportive and ECBI Problem suggesting that higher levels of adaptive and supportive ES skills were related to parents endorsing more behaviors as a problem prior to receiving treatment.

**Table 3.** Baseline Associations Between Parent and Child Measures of Interest.

Variable	ECBI intensity				ECBI problem			
	*B*	*SE*_B_	β	η_ρ_²	*B*	*SE*_B_	β	η_ρ_²
Intercept	66.53	45.98			.93	9.46		
DERS total score	−.19	.18	−.13	−.12	−.003	.04	−.01	−.01
CCNES non-supportive	5.60	1.89	.38	.32**	1.01	.40	.34	.29*
CCNES supportive	2.43	1.58	.20	.17	.67	.33	.28	.23*
Income	2.55	3.86	.08	.07	.64	.80	.09	.09

*Note.* DERS = Difficulties in Emotion Regulation Scale; CCNES = Coping With Children’s Negative Emotions Scale; ECBI = Eyberg Child Behavior Inventory.

* *p* < .05. **p < .01.

### Pre-to-Post Analyses

A hierarchical multiple regression was conducted to assess if baseline parent emotion regulation and socialization practices improved the prediction of children’s problem behaviors following the completion of treatment after controlling for baseline levels of child problem behavior (Table 4). There were no significant changes between each model, and the pre–post effect sizes were small.

**Table 4.** Associations Between Post-Assessment ECBI and Baseline ECBI, DERS, CCNES, and Income.

Variable	ECBI problem					ECBI intensity				
	Model 1		Model 2			Model 1		Model 2		
	*B*	β	*B*	β	η_ρ_²	*B*	β	*B*	β	η_ρ_²
Constant	3.19		*−*.22			51.28**		29.17		
Baseline ECBI	.41	.32	.42	.33	.32	.34	.41	.33	.40	.37
DERS			.01	.04	.03			.05	.04	.04
CCNES non-supportive			−.24	−.06	−.05			1.17	.10	.08
CCNES supportive			.05	.02	.01			.11	.01	.01
Income			.83	.99	.09			2.27	.08	.08
*R^2^*	.10		.12			.17		.18		
*F*	8.97		2.02			15.87		3.29		
*∆R^2^*	.10		.02			.17		.01		
*∆F*	8.97		.36			15.87		.29		

*Note.* DERS = Difficulties in Emotion Regulation Scale; CCNES = Coping With Children’s Negative Emotions Scale; ECBI = Eyberg Child Behavior Inventory.

**p < .01.

**Figure 1.**
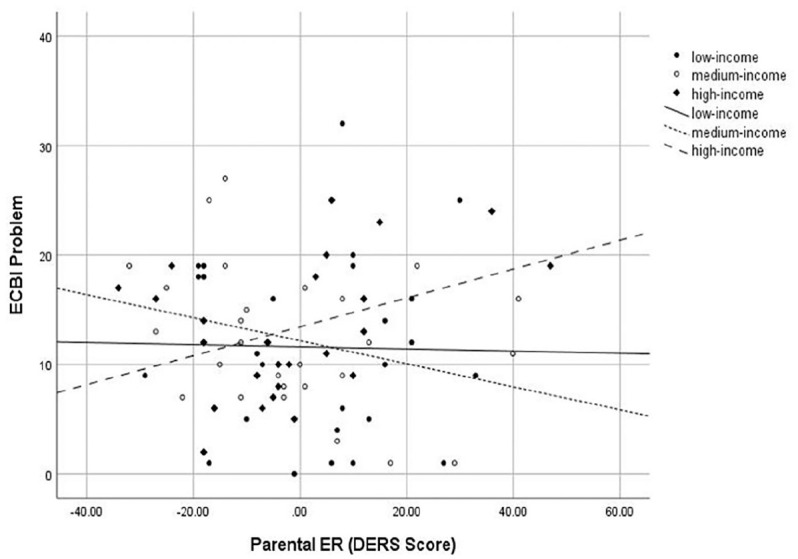
Interaction of baseline parental emotion regulation and income on post treatment ECBI Problem scores. *Note*. ER = emotion regulation; DERS = Difficulties in Emotion Regulation Scale; ECBI = Eyberg Child Behavior Inventory.

Based on previous theory regarding income and parental emotion regulation and socialization, analyses were completed to assess for any interactive effects of income by parent emotion regulation or socialization. No interactive effects were found for parent ER or ES regression models predicting post-assessment ECBI Intensity scores or for parental ES models predicting ECBI Problem scores. However, there was a significant interaction between income and parental ER for ECBI Problem Scores, *B* = 0.01, *t* = 2.34, *p* < .05, η_ρ_² = .25. The initial model including only children’s baseline ECBI Problem scores explained a significant proportion of variance in post-assessment scores, *R*^2^ = .10, *F*(1, 77) = 8.97, *p* < .01, while the model including parental characteristics and the interaction accounted for further variance, *R*^2^ = .18, *F*(5, 72) = 2.70, *p* = .02. The correlation between baseline parental ER and post treatment ECBI Problem scores was small (*r* = 0.019), moderate (*r* = 0.31), and high (*r* = 0.38) for low, middle, and high-income families, respectively (see Figure 1).

Paired-samples *t*-tests were completed to determine the pre- to post-treatment changes on the DERS, CCNES Non-supportive, CCNES Supportive and ECBI Intensity and Problem scores using Cohen’s *d* as a measure of effect size (*d* = 0.2 small, *d* = 0.5 medium, and *d* = 0.8 large; Cohen, 1988; Table 5). Parent’s maladaptive patterns of ES significantly decreased over the course of treatment (*t*(78) = 6.45, *p* < 0.01, *d* = 0.73) while more adaptive patterns of ES significantly improved over treatment (*t*(78) = −3.45, *p* < 0.01, *d* = 0.39). There was no significant change in parental ER over the course of treatment (*t*(78) = 0.69, *p* = .49, *d* = 0.07).

**Table 5.** Changes in Mean (*SD*), DERS, CCNES, and ECBI at Pre- and Post-Assessment.

Measure	Pre *M* (*SD*)	Post *M* (*SD*)	*t*	*df*	*p* Value	*d*
DERS	70.08 (17.97)	68.78 (19.82)	.69	78	.49	.07
CCNES non-supportive	8.28 (1.86)	7.30 (1.72)	6.45	78	.000**	.73
CCNES supportive	16.36 (2.29)	17.00 (2.04)	−3.45	78	.001**	.39
ECBI intensity	149.47 (27.47)	102.30 (22.69)	15.27	78	.000**	1.72
ECBI problem	22.63 (5.58)	12.35 (6.99)	12.35	78	.000**	1.39

*Note.* DERS = Difficulties in Emotion Regulation Scale; CCNES = Coping With Children’s Negative Emotions Scale; ECBI = Eyberg Child Behavior Inventory.

**p < .01.

Finally, to confirm that treatment was effective, children’s ECBI Intensity and Problem scores were examined. Both significantly decreased from pre- to post treatment (see Table 5).

## Discussion

This study explored the relationship between parental emotion regulation, socialization, and treatment response among families of young children who completed one evidence-based BPT program. Findings support guiding theories that parents’ emotional processes are linked to child problem behavior; although if and how these processes shape or are shaped by BPT may vary and in some cases depend on family income. While parent emotion regulation was not associated with child problem behavior at baseline, parents who reported greater emotion regulation difficulties also reported lower levels of supportive emotion socialization. This is consistent with prior work albeit largely in the internalizing literature as there has been less of a focus on ES and problem behavior generally (e.g., Bariola et al., 2011; Cole et al., 2003; Morris et al., 2007). In turn, parents who endorsed more maladaptive emotion socialization strategies also endorsed a greater occurrence and negative perception of problem behaviors at baseline. This pattern is consistent with prior work demonstrating that non-supportive emotion socialization strategies in particular may be associated with higher levels of child problem behavior at least concurrently (see Johnson et al., 2017, for a review). This echoes the long-standing theme in the family literature of “not being nasty matters more than being nice” or that “bad” experiences have a greater impact than “good” experiences (Baumeister et al., 2001; Ewart et al., 1991; Gonzalez et al., 2014). Consequently, clinicians may consider the importance of targeting parental emotion regulation as a mechanism to improve their supportive emotion socialization, decrease their maladaptive socialization patterns, and in turn, perhaps positively impact children’s behaviors.

Surprisingly, parents who endorsed higher levels of adaptive and supportive emotion socialization skills also reported that their children’s behavior was more (rather than less) of a problem at baseline. Although potentially counterintuitive, this pattern has been found before in the literature again highlighting the likely importance of “fit” (Grolnick et al., 1997; McElwain et al., 2007; Miller et al., 2015). Considering treatment-seeking families of young children with clinically significant problem behavior in particular, one possibility is that parents who experience their children’s behavior as more of a problem (i.e., higher ECBI Problem Score) start to use more adaptive and supportive emotion socialization strategies with the hope of helping the child to regulate their emotions and behavior. This makes intuitive sense; however, it also leads to the second hypothesis which would be to explore whether these efforts of reacting to the child’s emotions and behaviors could be functioning to inadvertently increase, rather than decrease, the problem behavior (i.e., increased attention). If this was the case, then efforts toward more flexible use of evidence-based treatment would suggest that clinicians may consider working with the parents to identify and actively model best practices in using adaptive and supportive emotion socialization strategies while also helping them to differentiate between appropriate expressions of emotions and disruptive behaviors. While standard BPT utilizes modeling to actively teach parents behavior management skills, it does not explicitly address modeling of parental ER or ES although this may be of particular importance in helping parents and children successfully progress through treatment and maintain positive outcomes. Specifically, clinicians may consider explicitly teaching and modeling ER skills for parents, particularly those with ER difficulties, to help them more successfully implement behavior management skills in times of stress (e.g., planned Ignoring, the Time-Out sequence). Future research may consider further investigating the role of clinician and parent-based modeling of ER and ES in the implementation of BPT. Additionally, the association between supportive ES and behavior problems was only found with regard to parents’ perceptions of behaviors as a problem (i.e., ECBI Problem Score), rather than their perceived level of the behavior (i.e., ECBI Intensity Score), which raises a third hypothesis. That is, perhaps parents who use more adaptive and supportive emotion socialization strategies are actually more bothered or frustrated by the behavior when it occurs or continues to occur in spite of their efforts. In this case, clinicians could begin to work with parents’ cognitions about and expectations for the child’s behavior in order to help them move away from how they think the child *should* respond and instead to focus on the child’s behavior and the ideal approach and timing of any strategy that is used, including supportive emotion socialization. Clinicians may work with families on ways to increase their use of supportive emotion socialization at times when children are more emotionally regulated (e.g., low-level whining while frustrated) and focus on implementing behavior management strategies when children display disruptive and problematic behaviors (e.g., screaming or yelling). Of note, such use of strategies may be most effective for children with clinically elevated problem behaviors and a different approach may be more appropriate for non-clinical samples. Finally, we consider a potential measurement explanation. That is, the CCNES was used as a measure of parental emotion socialization and only refers to strategies related to children’s feelings of sadness. Perhaps the various benefits and risks associated with supportive and unsupportive emotion socialization would be more explicit with measures that consider responses to aggression or other problem behavior in particular, given the primary presenting issues of this sample.

With these hypotheses in mind, it is also important to note that there were no significant associations between baseline supportive or non-supportive emotion socialization behaviors or parent emotion regulation and child problem behavior at post-treatment. This is again consistent with the conclusions of the Johnson et al. (2017) review of parent emotion socialization and child problem behavior literature which also found no prospective associations for supportive or non-supportive ES behaviors and child problem behavior in their meta-analysis. Johnson et al. (2017) did not include treatment outcome studies and posited that the lack of longitudinal associations may be a function of limited power; however, our findings suggest another possibility, which is that the role of parent emotion regulation and socialization may depend on other contextual variables such as family income. That is, emotion regulation (although not emotion socialization) seemed to be less of a critical ingredient for BPT outcomes for lower income families (i.e., parents reported similar levels of post-treatment behaviors regardless of their baseline levels of emotion regulation) relative to higher income families (i.e., parents reported higher levels of post-treatment problems with the child’s behavior relative to pre-treatment at higher levels of emotion dysregulation). However, it also important to consider additional contextual factors that may impact parents and children and are related to their income including neighborhood, parental education, and parental occupation. Future research may use a more nuanced examination of sociodemographic variables of the whole family system (e.g., multiple parents or caregivers), their relation to parental ES and ER, and the complex relationships between environmental and parental influences and children’s behavior problems (BeLue et al., 2015; Bronfenbrenner & Morris, 2007). Future work assessing these factors may help to provide insight into potential interactive or exacerbating effects among variables and suggest important additional targets of treatment.

Given theories of stress, distress, and the vulnerability for emotion dysregulation commonly discussed in the literature on financial strain, one potential explanation for the obtained pattern stems from finding this pattern with the problem score (i.e., how much of a problem is the behavior for the parent) but not the intensity score (i.e., how many problem behaviors the parent says are still occurring). Importantly, BPT provides concrete, behavioral skills to respond to children’s problem behavior and, in turn, can effectively reduce the frequency and intensity of that behavior. While the behavioral focus and strategies taught in BPT may be particularly well-suited for lower income parents, higher income families who are also more likely to have higher levels of education may further benefit from additional parent-focused cognitive work that more explicitly targets their expectations for their child’s behavior and, thus, how those expectations shape their perception of the behaviors as a problem (or not) when they occur (e.g., Luthar & Latendresse, 2005; Mah & Johnston, 2008; Shelleby & Shaw, 2014). Indeed, several studies of adolescents have highlighted the role of affluent parents’ expectations and focus on achievement and increased vulnerability for problem behavior (Luthar & Sexton, 2004; Randall et al., 2015). To this point, the standard course of BPT families received in this study led to parents’ reporting that they had experienced improved emotion socialization strategies, but no change in their own emotion regulation. Thus, discussions about enhanced BPT approaches may be most beneficial for higher income parents and/or those parents who may benefit most from strategies to regulate their own thoughts and feelings about whether the problem behavior “should” or “should not” be occurring and, thus, the degree to which is it is a problem.

There are several limitations to consider in the current study. First, the majority of participants consisted of mothers and their children. While female caregivers are more likely than male caregivers to attend appointments with their children in health care generally, there is a dearth of knowledge in current literature regarding the inclusion of fathers in studies implementing BPT in general as well as those targeting parental emotion regulation and socialization in BPT in particular (Labella, 2018). Furthermore, this study only collected information regarding parent’s marital status and did not collect information regarding potential co-parents in children’s lives. This is particularly important as the parent-child coercive cycle may vary both within and between families. In addition, this study did not include a measure of child emotion regulation, which has been implicated in the etiology, maintenance and treatment of child problem behavior (Bariola et al., 2011). For example, Chronis-Tuscano et al. (2016) reported that in addition to the promising effects of PCIT-Eco for child behavior in their uncontrolled case series design they also saw improvements in child emotion regulation. Interestingly, Salmon et al. (2014) also explored children’s emotion regulation in their comparison of standard Triple P and EETP; however, they found no differences between groups on children’s emotion knowledge. Third, all measures of parental emotion regulation, socialization and child behaviors were gathered by parent self-report. We found variability in patterns within and across these measures, suggesting that findings were not entirely due to social desirability or common method variance; yet, the inclusion of objective and observational measures may help to further elucidate patterns (Fischer et al., 2015; Morsbach & Prinz, 2006). Finally, the current study includes analyses of treatment completers only and did not employ intent-to-treat analyses; however, we viewed a focus on completers as appropriate given all families received an evidence based BPT program, HNC.

Although there are limitations, the current study possesses several strengths. First, this study used a treatment seeking sample that met clinical cut-off criteria on an established measure of child problem behavior to examine parent emotion regulation and socialization. Such a sample has received less attention than non-clinical samples as highlighted elsewhere (see Johnson et al., 2017, for a review). This is particularly important given calls in the literature to use findings from studies with non-clinical samples to draw conclusions about if and how BPT should be adapted based on patterns of ER and ES in families (England-Mason & Gonzalez, 2020; Johnson et al., 2017). In addition, consistent with increased emphasis on transdiagnostic mechanisms, including emotion regulation, this study focused on the role of parent emotion regulation and socialization in the treatment of children with a broad range of clinically significant problem behavior (Compas et al., 2017; Fernandez et al., 2016). Specifically, children in the current sample were diverse in clinical presentation of externalizing problems and all presented with behaviors that met clinical cutoffs for begin significantly above average. Third, in line with efforts to broaden empirical considerations of culture in the study of emotion regulation and socialization, as well as work emphasizing the culture of class (see England-Mason & Gonzalez, 2020; Hajal & Paley, 2020; Jones et al., 2018, for reviews), this study examined income as a moderator. Such work may begin to provide more direction regarding if and for whom adaptations to BPT are necessary or even critical. Fourth, this study included racially and ethnically diverse families, which is relatively rare in prior work on emotion regulation and socialization in general or studies of emotion regulation and socialization in the BPT literature (e.g., Labella, 2018; Mirabile et al., 2018; Rothenberg et al., 2019). Given the sample size and diversity both within and between families, we had limited power to examine ethnic or racial subgroups in meaningful ways, but this will be important for future work.

Turning to the broader implications of our findings, we acknowledge that they contribute to, rather than clarify, the mixed results characterizing this literature to date. While the inconsistent findings in the literature may result from variability in samples (e.g., clinical vs. community), methods (e.g., subjective vs. objective measures of ER, ES), and constructs (e.g., parent ER vs. parent ES vs. child ER), another possibility is that collectively we are missing the crux of what it means to measure emotional processes in families in general and in a treatment context like BPT in particular. That is, the bulk of research to date on emotion regulation, including this study, rely on trait-like, static, and individual measures using mean level analyses, which fail to adequately capture the nuanced and multifaceted aspects of emotion regulation and socialization processes in the dyadic or parent-child context as they relate to the etiology and treatment of early-onset behavior disorders in particular (Compas et al., 2017; England-Mason & Gonzalez, 2020). While this study investigated the impact of parental ER and ES on child behavior, it is likely that children’s behaviors play a role in shaping parents ER and ES and patterns of child behavior may make parents more or less likely to encounter difficulties with their own ER and supportive ES. Indeed, the coercive cycle suggests that it is the bidirectional processes between parents and children that contributes to the development and maintenance of problem behaviors over time. Although such a bidirectional approach is generally missing from current research in this area, methodological advances in work on other family-systems (e.g., parent and infants, couples), can perhaps begin to more substantively advance our understanding of the dynamic nature of these processes, including emotion coregulation, and BPT outcomes as well (Fischer et al., 2017; Gates et al., 2015; also see Gates & Liu, 2016; Zimmer-Gembeck et al., 2017, for reviews). Although a range of methods have been used to examine emotion regulation patterns in dyads, the more recent use of vocally encoded emotional arousal (e.g., fundamental frequency; f_0_) with couples, provides an objective, yet also feasible approach that may hold immense promise for the study of BPT as well (Fischer et al., 2015, 2017). Studies using f_0_ with couples have demonstrated its ability in assessing conflict, improvement in therapy, and risk factors in the dyad as well as the utility of such information for tailored treatment models (Baucom et al., 2009, 2012; Fischer et al., 2015, 2017). Thus, it may be imperative that future studies investigating the effects of emotion regulation and socialization in BPT begin to expand theory and methodology to include a more dyadic approach. Furthermore, future research implementing a bidirectional approach may help uncover more nuanced associations between parental ER, ES, and child behavior and provide some insight into more targeted treatment approaches for families with diverse presentations.
